# Remarkable Magnetic Properties in a Mn_73.6_Ga_26.4_ Alloy Produced via Out-of-Equilibrium Method

**DOI:** 10.3390/nano13233014

**Published:** 2023-11-24

**Authors:** Ovidiu Crisan, Alina Daniela Crisan

**Affiliations:** National Institute for Materials Physics, P.O. Box MG-7, 077125 Magurele, Ilfov, Romania; alina.crisan@infim.ro

**Keywords:** MnGa tetragonal phase, rare earth free magnets, magnetic properties

## Abstract

Rare-earth-free permanent magnets with the L1_0_ phase are actively researched for their potential as a future class of magnetic materials, capable of operating at higher temperatures and in challenging corrosion environments such as renewable energy applications. Among these classes, MnGa shows potential, being cost effective and having interesting magnetic properties. A MnGa magnetic alloy, with composition Mn_73.6_Ga_26.4_ in atomic percent, was produced via the out-of-equilibrium method, and its structural and magnetic properties were assessed using X-ray diffraction (XRD), transmission electron microscopy (TEM), selected area electron diffraction (SAED) and extended magnetic characterization. We show that the MnGa alloy submitted to thermal annealing in optimal conditions exhibits a two-phase microstructure, where small nanocrystals of tetragonal L1_0_/D0_22_ magnetic phase are embedded within a D0_19_ MnGa matrix of a non-collinear antiferromagnetic nature. These co-existing, magnetically different phases produce an optimal set of promising magnetic properties, larger than the values reported in the literature for single-phase MnGa alloys and thin films. Such large values are explained by the exchange coupling between competing non-collinear magnetic sublattices of the D0_19_ MnGa with the net moment of the small magnetic nanocrystals of tetragonal symmetry.

## 1. Introduction

Sustained efforts are being made nowadays in the search for new classes of rare earth (RE) free magnets, a search which is motivated by the continuous depletion of RE oxide resources and the need for improved magnetic parameters, especially for high-temperature applications. Indeed, RE-free magnets can operate in extreme conditions, such as high temperatures and corrosive media, which, for instance, is the milieu of operation for motors in wind turbines subjected to strong variations of temperature and humidity. Various possible RE-free magnets have been proposed and largely investigated, compounds deriving from the binary systems FePt [1], MnAl [2], MnBi [3] and others. What is common for all these different systems is the fact that they all may exhibit, in certain conditions, the formation of the tetragonal L1_0_ phase, which has been shown to present large magneto-crystalline anisotropy and high coercivity [1]. One of the potential solutions, FePt systems, is investigated in various shapes, for different applications. As nanoparticles obtained via chemical routes, FePt is highly bio-compatible being a good catalytic material. This system had been proposed as applicable for uses in hyperthermia therapy [4], magnetic resonance diagnosis [5] as well as for targeted drug delivery [6]. It has also been proposed for utilization as catalytic materials [7] or for sensing purposes [8,9]. For its fabrication, several different chemo-physical routes have been proposed. Among them, thermal decomposition has been found useful [10], as well as the polyol procedure [11], reverse micelles technique [12], microwave technology [13], self-assembly and sono-chemical preparation [14], liquid-based plasma technique [15], or condensation in the gas phase [16]. In the shape of bulk alloys, FePt systems can represent an alternative to present magnets in motors for wind turbines as they can, in principle, operate in tough conditions such as variable working temperature and corrosive environment. This is due to their performances: high Tc as compared to classical permanent magnets (470 °C), very large magneto-crystalline anisotropy (10^7^ MJ/m^3^) as well as high specific magnetization (1.4 T) [17,18]. At a larger scale, chemical synthesis and deposition techniques in controlled atmosphere or in high vacuum do not allow for a large yield of the material, as required for several applications; therefore, in this case, out-of-equilibrium synthesis pathways are the method of choice for such RE-free magnets. One of these methods is the rapid solidification from the melt, or melt-spinning [19]. It has been shown that the direct formation of the L1_0_ phase can be promoted in FePt alloys [19] by such out-of-equilibrium metallurgical methods, without the need for post-synthesis thermal annealing, as is needed currently in the case of nanoparticles or thin films. This is due to the ultrafast solidification from the melt, which can stabilize phases that are only possible in the liquid alloy, phases that are now accessible and attainable in the solid state. The method is particularly interesting in the case of MnGa system alloys, which constitute the object of the present paper. As another system, which can promote the formation of the L1_0_ phase, the MnGa binary system has quite complicated phase structure in the Mn-rich part of the phase diagram. Therefore, a method that can stabilize the L1_0_ phase to the detriment of other possible structures, such as the mentioned melt-spinning phase, seems more than appropriate for producing stabilized L1_0_ MnGa alloys. 

There is a unique combination of potential phases of magnetic interest in the Mn-Ga alloys, in the vicinity of the 70:30 Mn to Ga at% ratio. According to the binary phase diagram [20], there are three different possible crystal structures. The D0_19_-type Mn_3_Ga has a hexagonal crystal structure, is a non-collinear antiferromagnet and it is interesting for applications, such as exchange coupled interfaces, exchange bias and giant magneto-resistive materials [21]. The D0_22_-type Mn_3_Ga has a tetragonal crystal structure, is ferrimagnetic and it is interesting for spin polarization studies and spintronic devices. For instance, recently, spin orbit torque switching was documented in ferrimagnetic Mn_3_Ga [22]. Also, the ferrimagnetic phase has shown promising prospects in THz spintronics [23]. The L1_0_ MnGa phase is ferromagnetic, has high perpendicular magnetic anisotropy and shows good permanent magnetic properties, such as high coercivity and large-saturation magnetization, being considered as one of the potential classes of future RE-free permanent magnets [24,25]. It has been explained [26] that the peculiar type of magnetization in MnGa systems has its roots in the Mn atom electronic structure. Since the *d* shells of Mn atoms are half-filled, there is a certain flexibility in tuning their chemical environment and, therefore, their electronic properties are diverse, in a very narrow range of stoichiometry. The magnetic transition with the formation of L1_0_-MnGa was studied in MnGa alloy films, which were submitted to ion irradiation [27]. Here, it was shown that while the structure of the ion-irradiated films is made of L1_0_ ordered nanocrystals dispersed in an A1-disordered MnGa matrix, the magnetic anisotropy was not influenced by the process of irradiation, but the coercivity increased with the ion dose. Moreover, this increase was interestingly accompanied by an increased volume ratio of the disordered A1 to ordered L1_0_ MnGa phase, i.e., increased level of structural disorder in the alloy. 

The formation of L1_0_ phase MnGa was also evidenced in MnGa layers (52 to 60 at% Mn) grown via molecular beam epitaxy [28]. Here, isotropic in-plane magnetization was found to decrease with increasing Mn content, possibly due to competing interactions at the interface with the GaAs semiconductor surface. In another recent study, polycrystalline films of MnGa alloys grown on a CoGa buffer layer were shown to exhibit large perpendicular magnetic anisotropy and a maximum coercivity of 7 kOe [29].

The usefulness of the MnGa layered systems for various devices was also evidenced in [30], where a successful illustration of the spin torque diode effect in a magnetic tunnel junction in alternating layered systems MnGa/FeB/Fe was shown. Another useful effect of the MnGa was documented in [31], where L1_0_-MnGa films with an optimal coercivity of about 12 kOe were found to exhibit a strong anomalous Hall effect. Values of coercivity as high as 10 kOe were reported for a MnGa layer epitaxially grown on GaN(001) [32]. Being one of the most important magnetic properties, the coercivity and the maximum energy product are the main figures of merit for magnets in industrial applications; therefore, optimizing the material in terms of enhanced coercivity is an important direction of research in this field. Detection of the coercive field is of very high importance for magnetic materials. Theoretical tools, such as Monte Carlo simulation, successfully applied to ferromagnetic materials [33] are invariantly complemented by experimental determinations of coercivity and other magnetic parameters, for instance, by using vibrating sample magnetometry [34], very suitable for nanostructured magnetic materials. In what concerns the bulk MnGa systems, it has to be noted that high coercivity values have been found in bulk MnGa alloys (13.5 kOe), however, under magnetic annealing only [25]. 

In the present work, we report the formation of the L1_0_ and D0_22_ tetragonal phases in the MnGa, Mn-rich alloys synthesized by an out-of-equilibrium method, suitable for industrial applications. Moreover, we bring evidence for high magnetic performances for MnGa samples under various annealing conditions. We show that among other L1_0_-based magnetic materials, MnGa has promising prospects for applications in various industrial fields, being far more cost-effective than its FePt-based counterpart. 

## 2. Materials and Methods

The composition for the MnGa alloy was chosen to be situated in the Mn-rich part of the phase diagram, in order to better promote the magnetic features brought by the half-filled character of the *d* shell of Mn atoms. The chosen initial nominal composition of the alloy was, thus, 75:25 Mn to Ga ratio in at%, and it was fabricated as ribbons utilizing a melt-spun facility from Buehler (Buehler Melt Spinner MSP 10 from Edmund Buehler GmbH, Bodelshausen, Germany). For the synthesis, an intermetallic precursor was prepared by using small flakes of 99.99% high-purity Mn and Ga from Merck (Merck KGaA, Darmstadt, Germany). The preparation of the initial precursor involved melting in an induction oven under argon atmosphere (pressure 10^−1^ Torr). Obtained precursors were further re-melted three more times to produce a homogeneous enough alloy. After that, a quantity of 5 g of the precursor alloy was again re-melted inside of a small quartz crucible, resistively heated inside of the vacuumed chamber of the melt spinner. The crucible is dotted with a 3 mm nozzle located at the bottom of the crucible in close contact with the copper wheel for purging the melt. After achieving complete melting inside the crucible, an over pressure gas of about 70 kPa was used for purging out the melt through the nozzle, directly onto the surface of the fast-rotating copper wheel. The rotation frequency of the wheel was about 1200 rot/min, corresponding to a linear speed of around 28 m/s. This value corresponds roughly to a solidification rate of about 10^5^ K/min for the purged liquid, which is then transformed into long, cast ribbons of about 2.5 mm width and about 45 microns thickness. For inducing the occurrence of the L1_0_/D0_22_ phase, thermal annealing was performed in vacuum (10^−3^ Torr) in a resistive furnace. In order to accurately measure the real composition of the sample, the energy-dispersive X-ray spectroscopy (EDX) module of a scanning electron microscope Evo 50 XVP from Carl Zeiss NTS (Carl Zeiss Nano Technology Systems GmbH, Oberkochen, Germany) was used. The as-cast ribbons were submitted to two different annealing treatments at 400 °C and 500 °C, for 1 h, in order to promote structural ordering in the alloy. Microscopy imaging by TEM and high-resolution TEM as well as derived electron diffraction patterns (EDPs) were performed with the help of a JEM-ARM200F (from JEOL Ltd. Europe, Zaventem, Belgium) atomic resolution electron microscope, which operates at a 300 kV acceleration voltage. Structural studies were performed with the help of a D8 Advance diffractometer (Bruker AXS GmbH, Karlsruhe, Germany). XRD patterns were produced in a θ–2θ geometry between 15° and 90° (in 2θ), using the wavelength of the Cu Kα radiation (λ = 0.154 nm). A refinement method of the XRD data, compatible with full-profile Rietveld-type analysis, was performed on the obtained patterns using MAUD (Materials Analysis Using Diffraction) software (MAUD version 2.99, University of Trento, Trento, Italy). Magnetic measurements, i.e., initial magnetization as well as the hysteresis loops, were performed with the help of a SQUID (Superconducting QUantum Interference Device) using an MPMS (Magnetic Properties Measurement System) from Quantum Design (Quantum Design Europe GmbH, Darmstadt, Germany). The MPMS facility is extremely versatile and useful for detecting magnetic signal down to 10^−8^ emu. It has high resolution of up to 10^−11^ A m^2^ resolution and can provide an applied magnetic field of up to 12 T in a temperature interval between 2 and 400 K. The magnetic measurements were performed on as-cast and annealed samples up to 8 T applied field, oriented along the ribbons plane. The temperature of the measurements was set at 300 K. 

## 3. Results and Discussions

### 3.1. Structural Analysis by Diffraction

**Figure 1:**
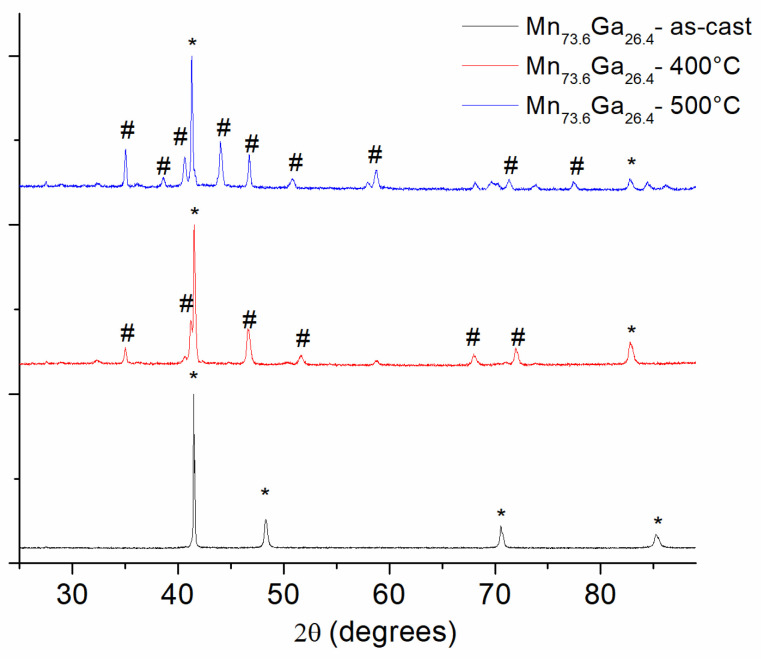
The X-ray diffraction patterns of the as-cast as well as annealed at 400 °C and 500 °C samples. The corresponding symbols are: *—D0_19_, #—tetragonal (L1_0_/D0_22_).

The energy-dispersive X-ray spectroscopy accurately revealed the real composition of the sample to be 73.6:26.4 Mn to Ga ratio (at%), within a 2% compositional error from the intended nominal composition. The structural analysis of the as-cast and annealed Mn_73.6_Ga_26.4_ samples was performed using both XRD and high-resolution TEM, which, in turn, also provided the electron diffraction patterns, and the two results were further compared and assessed. Figure 1 shows the XRD patterns of the as-cast and the two annealed samples. The as-cast sample provides the image of a single-phase character. The observed Bragg lines, marked with * on the graph, were identified by the full-profile analysis as belonging to the hexagonal D0_19_ Mn_3_Ga phase. Being one of the most stable in the MnGa phase diagram [20], it was expected that we would find it here, as the as-cast structural precursor. D0_19_ Mn_3_Ga is hexagonal, having a Mg_3_Cd-type structure. The space group is *P*6_3_/*mmc* and the lattice constants, as determined from the full-profile analysis, are as follows: *a* = *b* = 5.692 Å, *c* = 4.537 Å. In the D0_19_ Mn_3_Ga, the manganese atoms form a kagome-type lattice in the basal planes, which further stack along the *c* axis, where Ga atoms are located in the center of Mn atom hexagons [21]. For the annealed samples, the image is rather different. More Bragg lines appear, giving hints about a second crystalline phase occurring in the samples upon annealing (Figure 1). Starting with 400 °C annealing, the pattern shows additional Bragg peaks. For the indexation of the phase occurring after annealing, several aspects must be discussed. It has been argued [24] that Mn_3−x_Ga is a continuous solid solution, of tetragonal symmetry, with a range decreasing x from 0 to 1, respectively, from Mn_3_Ga (D0_22_) to Mn_2_Ga (L1_0_). D0_22_ can be regarded as a highly distorted variant of the cubic L2_1_ Heusler structure, XYZ_2_, where X, Y and two Z atoms occupy four interpenetrating face-centered cubic sublattices [24]. D0_22_ structure is very similar to the tetragonal L1_0_, with alternate layers of ordered X and Y atoms and two Z atoms, where X = Ga and Y, Z = Mn. The L1_0_ structure is obtained when the X and Y atoms are identical (Ga). Both being tetragonal, the D0_22_ phase crystallizes in *I*4/*mmm* space group and it is encountered for Mn content between 63 and 81%, and L1_0_ phase crystallizes in the *P*4*/mmm* and is encountered for Mn content between 50 and 70% [35]. According to [35] and also to the MnGa phase diagram [20], there could be a range of Mn content where these two tetragonal phases could co-exist. Their distinctive signature in XRD patterns is the occurrence of the so-called superlattice peaks. It is to be noted that while the *c* parameter of the D0_22_ is basically the double of the *c* parameter of the L1_0_ phase, the *a* parameter does not vary significantly. In addition, the angular positions of the two superlattice peaks, as shown by Niida et al. [25], the (001) and (110) Bragg reflections, are very similar. 

We were able to index on the patterns recorded for the samples annealed at 400 °C and 500 °C the superlattice peaks (001), (011), (101) and (110), belonging to the tetragonal MnGa phase. Upon increasing the annealing temperature, at 500 °C annealing, the patterns show even more pronounced superlattice peaks with heightened intensity. Since the sole XRD pattern cannot give distinct proof of either of the two tetragonal phases, we infer that these additional peaks, marked with # on the graph belong to either of the two D0_22_ and L1_0_ tetragonal MnGa phases. The only indication at this point for tipping the scale in favor of one or the other is represented by the Mn content, which is, in fact, close to the border between L1_0_ and D0_22_. We proceeded to a full-profile analysis of the XRD patterns by using MAUD software, and both of the patterns for the samples annealed at 400 °C and 500 °C could be accurately fitted with one tetragonal D0_22_ Mn_3_Ga phase, *I*4/*mmm* space group (PDF file: 04-015-2490) and with one tetragonal L1_0_ Mn_2_Ga phase, *P*4/*mmm* space group (PDF file: 03-065-6327). The lattice parameters of the two phases are listed in Table 1.

These structural results and the consequent phase structure in the annealed samples showed further confirmation in the following section of the paper, dedicated to the electron microscopy analysis. 

### 3.2. High-Resolution TEM

**Figure 2:**
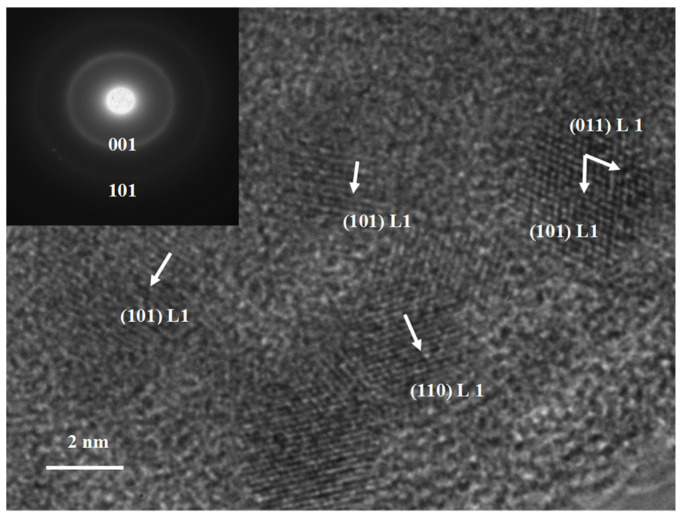
High-resolution transmission electron micrograph of the sample annealed at 500 °C. The inset is showing the corresponding electron diffraction pattern of the TEM image.

The structural data obtained and discussed in the previous section gain further consolidation with the structural analysis performed using TEM and the electron diffraction patterns. The sample annealed at 500 °C was imaged using TEM, and one obtained image, relevant for its structure, is presented in Figure 2. In this high-resolution image, small crystallites are observed in the microstructure crystallites, which are between 2 and 5 nm in size. These crystallites are immersed in a residual matrix of a different crystal structure. The high-resolution image allowed for the observation of atomic planes within the small nanocrystals. The interplanar distances, or *d*-spacings, between these observable atomic planes were accurately measured and found to be consistent with several of the *hkl* reflections, corresponding to the superlattice lines of the tetragonal L1_0_ phase. As such, measuring the *d*-spacings allowed us to unambiguously identify the (001), (011) and (101) reflections belonging to the L1_0_ tetragonal symmetry. In the inset, the corresponding electron diffraction patterns are shown, mapping the reciprocal space of the TEM image. Two weak rings are observed and accurately measured on the pattern. The measuring allowed us to determine that they belong to the (001) and (101) reflections of the tetragonal L1_0_ phase, respectively. It is, therefore, concluded that, in complete agreement with the observations from the XRD diffractograms, the phase structure of the MnGa sample annealed at 500 °C contains, in addition to the already found tetragonal D0_22_, a non-negligible amount of tetragonal L1_0_ phase, which are phases of interest for potential applications of the MnGa system as a future RE-free magnetic material. 

### 3.3. Magnetic Characterization

**Figure 3:**
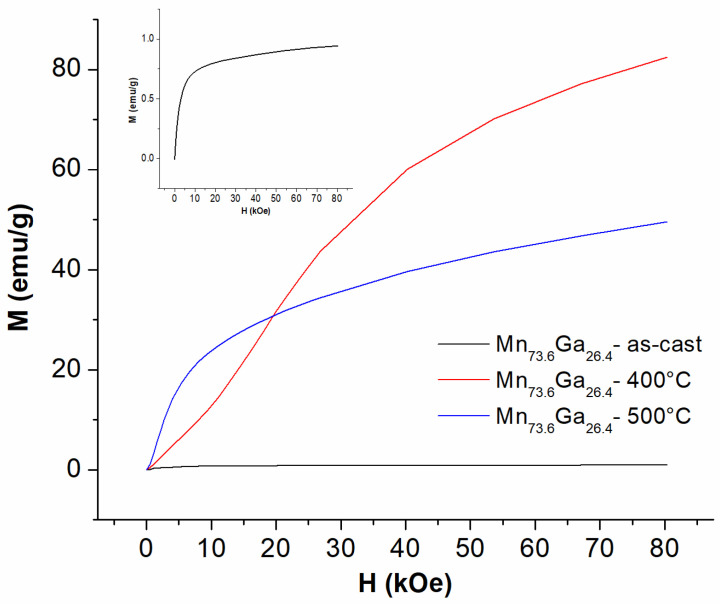
300 K initial magnetization recorded under an applied field of up to 8 Tesla, for the as-cast sample, as well as for the samples annealed at 400 °C and 500 °C. The field is applied parallel to the ribbons plane. Inset: plot at smaller scale of the magnetization of the as-cast sample.

The magnetic characterization of the samples was performed using SQUID magnetometry. The first measurement protocol consisted of mounting and centering the sample within the measurement coil. Then, the initial magnetization of the samples was recorded, without any previous orientation of the samples, by applying a magnetic field. This is used to preserve the pristine character of the samples, allowing us to accurately observe the behavior of the magnetic moments in the applied magnetic field, without any previous history of magnetization. Each of the SQUID measurements was taken on one piece of ribbon, 2 mm × 2 mm × 0.045 mm. This piece was fixed onto the sample holder, with the applied magnetic field parallel to the ribbon’s plane. The weight of the ribbon piece measured was about 1.2 mg. Demagnetization factor of the sample was calculated assuming the size of the ribbon piece measured in SQUID (2 mm × 2 mm × 0.045 mm) to be around 1.5 × 10^−4^, following the model developed for magnetic melt-spun ribbons by D.N. Zhmetko [36]. Corrections for the demagnetization factor were carefully applied before plotting the magnetization results. The initial magnetization, recorded for all three samples, as cast and annealed at 400 °C and 500 °C, is shown in Figure 3.

It can be seen that the initial magnetization value for the as-cast sample shows very low values of magnetization, around 1 emu/g, as seen in the inset. The approach to saturation evokes a very weak ferrimagnetism where there is a fast approach to saturation; however, the saturation values are much smaller than the annealed samples. This result is consistent with the structural data, which showed that in the case of the as-cast sample, the hexagonal D0_19_ MnGa is present and predominant. 

There is, however, a completely different behavior in the case of the two annealed samples. It can be seen that upon applying the magnetic field, the initial magnetization increases consistently, as is the case in the ferromagnetic materials. While the increase in the initial magnetization about the presence of a ferrimagnetic fraction in the sample, the lack of saturation of the initial magnetization provides further evidence for the presence of the other crystalline phase, remaining in the annealed samples, as seen in the XRD patterns. Furthermore, it should also be noted that the sample annealed at 400 °C has a slower approach to saturation than in the case of the sample annealed at 500 °C, but its initial magnetization increases towards much higher values at higher applied magnetic fields than its counterpart. This shows that there could be an optimum of the annealing conditions for optimal magnetic properties, as annealing at higher temperatures might be detrimental for the overall magnetic properties. These assumptions are further confirmed upon analyzing the hysteresis loops for the three samples, as-cast and annealed. 

**Figure 4:**
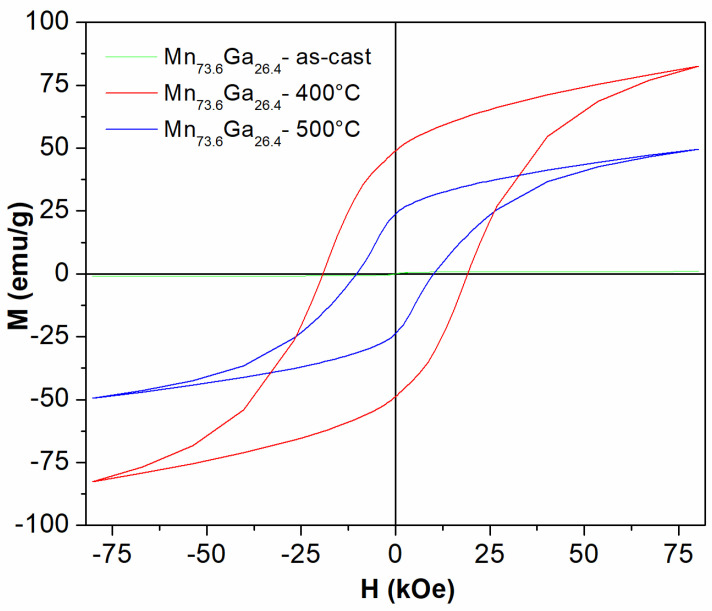
300 K hysteresis loops recorded under an applied field of up to 8 Tesla, for the as-cast MnGa sample, as well as for the samples annealed at 400 °C and 500 °C. The field is applied parallel to the ribbons plane.

The hysteresis loops recorded at 300 K under a magnetic field applied parallel to the ribbon plane of up to 8 Tesla are imaged in Figure 4. As was the case of the initial magnetization curve, the as-cast sample shows very small net magnetization upon increasing the applied field. On the contrary, the two annealed samples show a strong increase in both net magnetization and coercivity. Again, as was the case of the initial magnetization curves, the two annealed samples show no saturation, even at the highest applied field. The sample annealed at 400 °C exhibits a maximum specific magnetization of about 81 emu/g, while the sample annealed at 500 °C shows a smaller value of the maximum specific magnetization of about 49 emu/g. Taking into account the density of the sample (6.69 g/cm^3^), we calculated the maximum magnetization to be around 0.54 M A/m for the sample annealed at 400 °C and 0.32 M A/m for the sample annealed at 500 °C, respectively.

It is known that in the bulk MnGa alloys, the magnetization increases steadily as the Mn content decreases from 75 at% to 66 at% [37]. This is explainable by the so-called manganese dilemma, because in order to obtain a large moment, the Mn atoms have to be fairly well separated, which, at the same time, decreases the magnetization [24] as this parameter is calculated as the magnetic moment per unit volume. This also suggests a change in Mn site occupancy. On decreasing the Mn content below 75 at%, towards forming a Mn_2_Ga phase, more and more Mn atoms will migrate to the 4d sites in the tetragonal unit cell, which increases the magnetization compared to the Mn_3_Ga. In our case, the phase structure of the annealed samples contains both the L1_0_ Mn_2_Ga tetragonal phase as well as the Mn_3_Ga D0_22_ tetragonal phase in various proportions, and this produces the high value of magnetization of 0.54 M A/m. This is influenced, on the other hand, by the exchange interaction between the tetragonal nanograins and its strong dependence on interatomic Mn–Mn distances that mainly governs the arrangement of magnetic Mn-moments and, hence, the magnetization of the alloys. Indeed, it has been shown [24] that Mn atoms on sites with the shortest Mn–Mn distances, <240 pm, tend to be nonmagnetic, those on sites distanced between 250 and 280 pm tend to couple antiferromagnetically and only Mn atoms on sites with the longest bonds, >290 nm, tend to have larger moments, ferromagnetically coupled. The value we obtained is still below the maximum predicted value of 0.7 M A/m in Mn_2_Ga if all Mn atoms would occupy the 4d sites of the tetragonal unit cell, retaining their moment of 2.08 µB [24]. However, our value of 0.54 M A/m is higher than the values of about 0.47 M A/m encountered in thin films of Mn_3_Ga [38]. This result is in agreement with the suggestion formulated in [24] that there is room for improvement and optimization of magnetic performances around areas with D0_22_ structure or in the regions in the Mn–Ga phase diagram where the L1_0_ phase appears. Similarly good results in systems involving D0_22_ phase were also obtained in the case of the MnGe system [39,40] 

The most remarkable results are represented by the very large values of the coercive field and of the remanence shown for the two annealed samples. It can be seen that while the sample annealed at 500 °C exhibits a coercive field of 12 kOe and a remanence value of about 25 emu/g, the sample annealed at 400 °C shows even higher values of coercivity and remanence: 19.7 kOe and 48 emu/g, respectively. These values exceed the results obtained in MnGa thin films by far, as well as in other MnGa bulk alloys, with lower Mn content, reported in the literature, and the highest value encountered in the literature for MnGa binary system being 13.5 kOe [25]. These high coercive values can be attributed to the exchange coupling between the two tetragonal phases present in the samples, as well as the coupling between the non-collinear antiferromagnetic, residual D0_19_ MnGa phase and the tetragonal nanograins. It is also to be noted that there is a slight exchange bias effect of about 0.37 kOe for the sample annealed at 400 °C, an effect which is due, in fact, to the competing interactions between the non-collinear sublattices of the antiferromagnetic D019 phase with the highly abundant tetragonal ferrimagnetic phases. Moreover, the occurrence of the exchange bias effect also proves unequivocally that there is still a small antiferromagnetic fraction in the annealed samples, confirming once again the structural characterization results. It is inferred that the competing effect of the D0_19_ magnetic sublattice exchange coupled with the magnetic moments of small nanocrystals of tetragonal phases might give these large values of remanence and coercivity. However, these findings need further clarification. As such, a more detailed study involving several more MnGa alloys with different stoichiometries is underway, on the quest to explain these large values of magnetic properties. 

## 4. Conclusions 

We produced a Mn_73.6_Ga_26.4_ magnetic alloy via an out-of-equilibrium method, and we succeeded in stabilizing the co-existence of two competing, magnetically different, structural phases. Extended structural analysis by XRD, TEM imaging and EDP showed that the MnGa alloy submitted to thermal annealing in optimal conditions has a microstructure, where small nanocrystals of tetragonal L1_0_ with low abundance co-exist with the tetragonal D0_22_ ferrimagnetic phase, which is the most abundant in the samples annealed at 400 °C and 500 °C, respectively. Moreover, there is a residual D0_19_ MnGa matrix, with a lower content of a non-collinear antiferromagnetic nature. These co-existing, magnetically different phases produce an optimal set of promising magnetic properties, with very a high coercive field accompanied by a large enough remanence, higher than the values reported in the literature for single-phase MnGa alloys and thin films. Such large values are explained by the exchange coupling between competing non-collinear magnetic sublattices of the D0_19_ MnGa with the net moment of the tetragonal ferrimagnetic L1_0_ and D0_22_ nanocrystals. These findings are to be confirmed and correlated in further studies, involving MnGa alloys with different stoichiometries, in an attempt to confirm our hypotheses.

## Figures and Tables

**Table 1:** Lattice parameters for the two tetragonal phases.

Sample	D0_22_^1^	L1_0_^1^	Abundance ^2^
Mn_73.6_Ga_26.4_ annealed at 400 °C	a = 3.891 Å c = 7.072 Å	a = 3.879 Å c = 3.582 Å	D0_22_: 58% L1_0_: 27%
Mn_73.6_Ga_26.4_ annealed at 500 °C	a = 3.886 Å c = 7.064 Å	a = 3.876 Å c = 3.579 Å	D0_22_: 72% L1_0_: 12%

^1^ Errors in the lattice parameters determination: ±0.003 Å to ±0.007 Å. ^2^ Errors in the abundance determination: ±1.4% to ±2.3%.

## Data Availability

The data presented in this study are available on request from the corresponding author. The data are not publicly available due to IPR protection measures.
